# Data on producing an infusion fluid that contains nitric oxide

**DOI:** 10.1016/j.dib.2019.105011

**Published:** 2019-12-17

**Authors:** Yoshihiro Tange, Shigenori Yoshitake, Wataru Watanabe

**Affiliations:** Department of Medical Engineering, Kyushu University of Health and Welfare, Nobeoka City, Japan

**Keywords:** Nitric oxide, Biocompatibility, Intravenous infusion fluid, Data

## Abstract

Nitric oxide (NO) is a vasodilator and platelet aggregation inhibitor. In patients with pulmonary hypertension, inhalation of NO is used as a therapeutic option. It has been proposed that nitrite (NO_2_^−^) is a constitute intravascular storage and delivery source of NO, a potent cardioprotective-signaling molecule. The administration of NO_2_^−^ could have therapeutic effects in conditions where the oxygen-dependent enzymatic production of NO is compromised (i.e., ischemia). Thus, if NO could be supplied by an intravenous infusion fluid, it would be an easier method than by inhalation or delivery to the blood vessels by the blood stream. We produced 2 types of solutions, i.e., a nitrogen gas injected solution (control solution) and NO gas injected solution (experimental solution). NO was measured by the Microplate Photometer (MultiSkan FC, Thermo Fisher Scientific K.K., Tokyo, Japan) with a 540-nm wavelength and NO assay kit (Quantichrom™ Nitric Oxide Assay Kit, BioAssay Systems, Hayward, CA, USA). Gas profiles were measured by the EG6+ (Abbott Japan Co., Ltd., Osaka, Japan) with an i-STAT system (300F, Abbott Japan Co., Ltd.). Comparisons of gas profiles and measured NO concentrations in vitro and ex vivo are shown between the control and experimental solutions. Since NO is oxidized to NO_2_^−^ and nitrate (NO_3_^−^), it is common practice to quantitate total NO_2_^−^/NO_3_^−^ as a measure of the NO level. We used the assay that was designed to accurately measure NO production following reduction of NO_3_^−^ to NO_2_^−^ using the Griess method. The data in this document describe production of an infusion fluid that contains NO without any special devices.

**Table:** Specifications table

Subject	Medicine
Specific subject area	Biotechnology
Type of data	Table Figure
How data were acquired	Nitric oxide (NO) was measured by the Microplate Photometer (MultiSkan FC, Thermo Fisher Scientific K.K., Tokyo, Japan) with a 540-nm wavelength and NO assay kit (Quantichrom™ Nitric Oxide Assay Kit, BioAssay Systems, Hayward, CA, USA). Gas profiles were measured by the EG6+ (Abbott Japan Co., Ltd., Osaka, Japan) with an i-STAT system (300F, Abbott Japan Co., Ltd.).
Data format	Raw Analyzed
Parameters for data collection	Infusion fluid (Sublood BSG, Fuso, Osaka, Japan) was used as the test solution. One thousand mL of nitrogen gas (control solution) or 1000 mL of NO gas with 1000 ppm was injected into 2020 mL of the supplemental fluid (experimental solution). Bovine blood was obtained from a local distributor.
Description of data collection	Analysis of the gas profiles of the control and experimental solutions included the pH, partial pressure of carbon dioxide, partial pressure of oxygen, base excess, bicarbonate, sodium, and potassium. NO levels were measured in the solutions and bovine blood.
Data source location	Department of Medical Engineering, Kyushu University of Health and Welfare, Nobeoka City 8828508, Japan
Data accessibility	Data are provided as supplementary Excel tables within the article.

**Table:** 

**Value of the Data** • A solution containing NO is able to be produced by injecting NO gas into an infusion fluid. • NO-containing fluid is able to be produced quite simply without any special devices. • NO can be delivered to patients in whom biological responses to NO are needed via intravenous infusion.

## 1 Data description

**Fig. 1:**
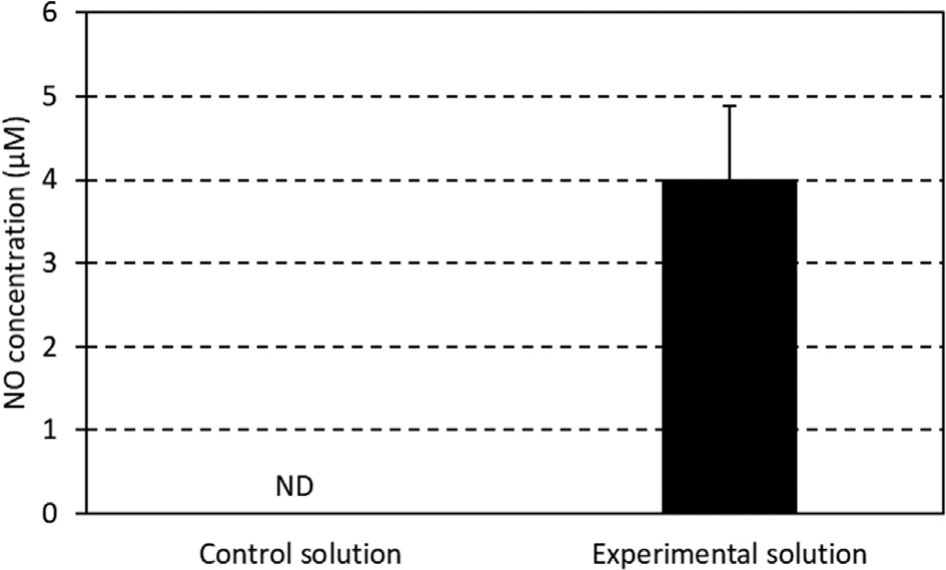
Concentrations of nitric oxide between the control and experimental solutions (n = 3). Data are expressed as mean ± standard deviation. ND, not detected.

The data presented here shows production of a solution that contains NO with reference to an article [1]. The supplemental fluid (Sublood BSG, Fuso) was used as the test solution. One thousand milliliters of nitrogen gas (control solution) or 1000 mL of NO gas with 1000 ppm was injected into 2020 mL of the supplemental fluid (experimental solution). Gas profiles of the control and experimental solutions were determined immediately after opening the solutions. The data are shown in Table 1. NO levels in both solutions are shown in Fig. 1. NO concentration in the blood diluted by the control and experimental solutions are shown in Table 2. Tables and figure are creating from supplementary file within the article.

**Table 1:** Comparison of gas profiles between the control and experimental solutions (n = 6).

	Control solution	Experimental solution
pH	7.26 ± 0.02	7.28 ± 0.05
PCO_2_ (mmHg)	71.0 ± 4.6	67.2 ± 8.8
PO_2_ (mmHg)	53.7 ± 1.2	56.0 ± 2.2
BE (mmol/L)	4.7 ± 0.5	5.0 ± 0.9
HCO_3_^−^ (mmol/L)	31.8 ± 0.5	31.6 ± 0.6
Na (mmol/L)	141.7 ± 0.5	142.0 ± 0.0
K (mmol/L)	1.9 ± 0.0	1.9 ± 0.0

Data are presented as a mean ± standard deviation.

PCO_2_, partial pressure of carbon dioxide; PO_2_, partial pressure of oxygen; BE, base excess; HCO_3_^−^, bicarbonate; Na, sodium; K, potassium.

**Table 2:** Concentrations of nitric oxide in the blood diluted by the control and experimental solutions (n = 6).

Diluted solution	Twice	Threefold
Control solution (μM)	3.44 ± 0.70	3.84 ± 3.24
Experimental solution (μM)	6.22 ± 1.80	7.09 ± 1.75

Data are presented as a mean ± standard deviation.

## 2 Experimental design, materials, and methods

### 2.1 Fluid samples

Fluid samples were prepared using a conventional bicarbonate supplemental fluid (Sublood BSG, Fuso); the air was removed using a syringe (Nipro, Osaka, Japan). We prepared two types of fluids. One thousand mL of nitrogen gas (control solution) or 1000 mL of NO gas with 1000 ppm was injected into 2020 mL of the supplemental fluid (experimental solution). The comparison of gas profiles between the control and experimental solutions is shown in Table 1.

### 2.2 Analysis of the NO concentration in the control and experimental solutions

We measured the NO concentration at 60 minutes after injecting gas into the supplemental fluid in both solutions. NO was measured by the Microplate Photometer (MultiSkan FC, Thermo Fisher Scientific K.K.) with a 540-nm wavelength using the NO assay kit (Quantichrom™ Nitric Oxide Assay Kit, BioAssay Systems) (Fig. 1.). NO was measured according to the protocol of the assay kit. It is common practice to quantitate the total NO_2_^−^/NO_3_^−^ as a measure of the NO level.

### 2.3 Blood samples

Bovine blood was obtained from a local distributor, and 3.2% of sodium citrate (Wako Pure Chemical Industries, Ltd., Osaka, Japan) was used as an anticoagulant. Mean hematocrit and hemoglobin values were 28% and 9.5 g/dL, respectively.

### 2.4 Analysis of the NO concentration in the blood diluted by the control and experimental solutions

We prepared 100 mL of bovine blood in infusion bags made of polyethylene (Fuso). The blood was diluted twice (100 mL) and threefold (200 mL) by the control or experimental solution. The infusion bags were shaken manually for 1 minute and placed on a table until the bubbles visually diminished. NO was measured by the Microplate Photometer with a 540-nm wavelength using the NO assay kit (Table 2). NO in the blood was added to the deproteinization treatment and measured according to the protocol sheet in the assay kit. All experiments were completed within 12 hours after obtaining the blood samples.
